# The MRI of Lumbar Vertebral Canal in Low Back Pain: A Cross-Sectional Study

**DOI:** 10.7759/cureus.51407

**Published:** 2023-12-31

**Authors:** Asim Mohsin Badaam, Shivaji B Sukre, Syed Sajjad Ali Hashmi, Siraj Ahmed Hasham Shaikh, Sanket Dadarao Hiware, Khwaja Moizuddin Jawaduddin, Syed Rehan H Daimi, Faiza Banu Siddiqui, Mohammed Taher Ali, Khaled M Badaam

**Affiliations:** 1 Department of Anatomy, College of Medicine, Imam Abdulrahman Bin Faisal University, Dammam, SAU; 2 Department of Anatomy, Government Medical College (GMC), Parbhani, IND; 3 Department of Radiology, Jamia Islamia Ishaatul Uloom's (JIIU) Indian Institute of Medical Science and Research (IIMSR), Jalna, IND; 4 Department of Anatomy, Graphic Era Institute of Medical Sciences, Dehradun, IND; 5 Department of Pharmacology, College of Clinical Pharmacy, Imam Abdulrahman Bin Faisal University, Dammam, SAU; 6 Department of Physiology, Government Medical College (GMC), Aurangabad, IND

**Keywords:** low back pain (lbp), lumbar vertebra, spinal mri, lumbar spinal canal stenosis, vertebral canal

## Abstract

Background

The imaging of the lumbar canal is an important aspect of low back pain (LBP) management. Magnetic resonance imaging (MRI) has gained widespread acceptance for the evaluation of spine anatomy.

Objective

The objective of the study is to compare the MRI findings of the anteroposterior diameter, transverse diameter, and thecal sac area of the lumbar vertebral canal in symptomatic low back pain patients to that of patients without low back pain.

Methods

The cross-sectional study included 200 subjects of which 100 subjects (49 males and 51 females) were symptomatic cases of low back pain and 100 subjects (53 males and 47 females) had no symptoms of low back pain and were enrolled as controls. The MRI scans were studied for the anteroposterior diameter, transverse diameter, and thecal sac area of the lumbar vertebral canal.

Results

In our study, the anteroposterior diameter of the lumbar vertebral canal among cases was found to have a mean of 14.42, 14.09, 13.44, 13.63, and 13.79 with a standard deviation (SD) of 1.25, 1.32, 1.75, 1.75, and 2.65 at L1, L2, L3, L4, and L5 levels, respectively. The anteroposterior diameter of the lumbar vertebral canal among controls was found to have a mean of 15.26, 15.16, 14.71, 14.68, and 15.28 with an SD of 1.60, 1.67, 1.30, 1.36, and 1.97 at L1, L2, L3, L4, and L5 levels, respectively. The difference in anteroposterior diameters of the lumbar vertebral canal was found to be statistically significant at each level, between cases and controls. The transverse diameter of the vertebral canal was found to be smaller in cases as compared to controls with a statistically significant difference at each of the levels studied. The thecal sac area of the vertebral canal was found to be less in subjects with low back pain at each of the vertebral levels studied.

Conclusion

The study results provide insight into the lumbar vertebral parameters in the study population and give comparative data among the symptomatic low back pain patients and control subjects without low back pain. The MRI reflected decreased anteroposterior diameter, transverse diameter, and thecal sac area of the lumbar vertebral canal among symptomatic low back pain patients.

## Introduction

Low back pain (LBP) is defined by its location, typically between lower rib margins and buttock creases. LBP is often associated with pain in one or both legs, and some of the LBP patients have neurological symptoms in the lower limbs. LBP has been reported to affect up to two-thirds of adults at some point in their lifetime [1-4]. Spinal stenosis is the narrowing of the central spinal canal. Lumbar spinal stenosis may be caused by the lumbar vertebral canal or foramina narrowing due to multiple factors, i.e., degenerative changes such as facet osteoarthritis, hypertrophy of the ligamentum flavum, and bulging discs. An anteroposterior diameter of the lumbar vertebral canal of less than 12 mm is considered a strong indicator of stenosis [1-6]. Spinal stenosis is among the most frequent reasons for advising spine imaging. Magnetic resonance imaging (MRI) is the preferred investigation as it is noninvasive and capable of multiplanar imaging [6-10]. The present study evaluates the anteroposterior diameter, transverse diameter, and thecal sac area of the lumbar vertebral canal among symptomatic low back pain patients visiting the tertiary care center in India's Marathwada region of Maharashtra.

## Materials and methods

**Figure 1:**
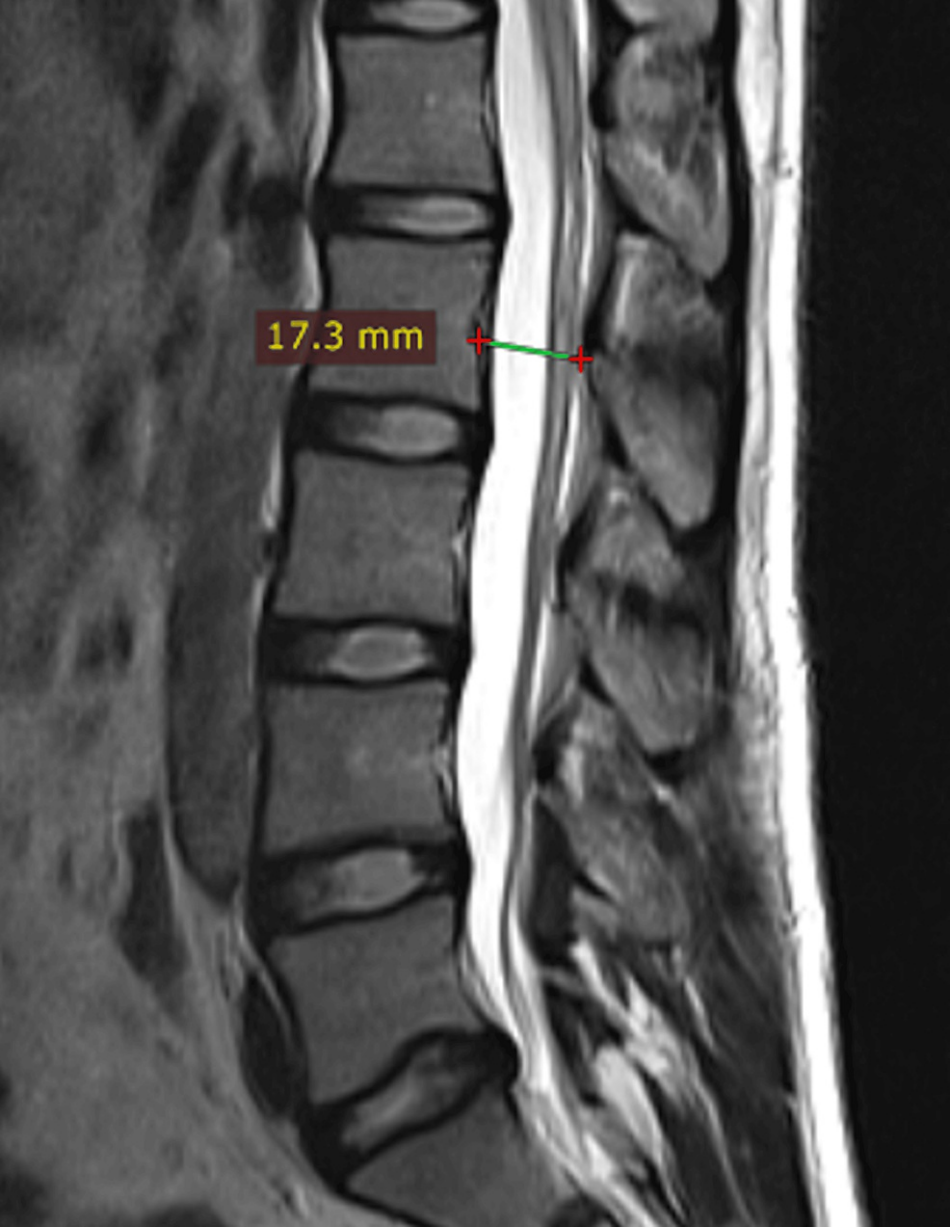
The measurement of the anteroposterior diameter of the lumbar vertebral canal

The present cross-sectional study was conducted at a tertiary care center in the Marathwada region of Maharashtra, India. The Institutional Ethics Committee of Government Medical College, Aurangabad, approved the study protocol (approval number: IEC-GMCA/526/2012), and informed consent was obtained from each study participant. Lumbar MRI scans were performed from January 2013 to November 2014 among patients undergoing lumbar spine MRI in the age group of 21-80 years, with no history of obvious lumbar congenital spine abnormality or lumbar spine metastasis. Patients with congenital disorders of the lumbar spine, acute lumbar spine injury, and lumbar spine metastasis and individuals with metallic implants were excluded from the study. The study included 200 subjects, of which 100 subjects (49 males and 51 females) had symptomatic low back pain and were labelled as cases and 100 subjects (53 males and 47 females) had no symptoms of low back pain and were enrolled as controls. The MRI scans were studied for the anteroposterior diameter of the lumbar vertebral canal. MRI scan was performed with the "Philips MR Achieva" (Amsterdam, Netherlands) 1.5 tesla scanner. The adjusted slice thickness was 4 mm with sequence T1-weighted (T1W) fast spin echo (FSE) sagittal (SAG), T2-weighted (T2W) FSE SAG, T1W FSE axial, T2W FSE axial, and T2 FSE drive axial (high resolution) using drive with a small field of view (FOV) to reduce cerebrospinal fluid flow artifacts. Measurements were recorded using the General Electric software (Boston, MA). The anteroposterior diameter of the lumbar vertebral canal was measured anteroposteriorly in the midsagittal plane from the mid-point of the posterior surface of the vertebral body to the mid-point of the inner bony margin of the posterior arch (Figure 1). The mean, range, and standard deviation (SD) were calculated for cases and controls separately.

**Figure 2:**
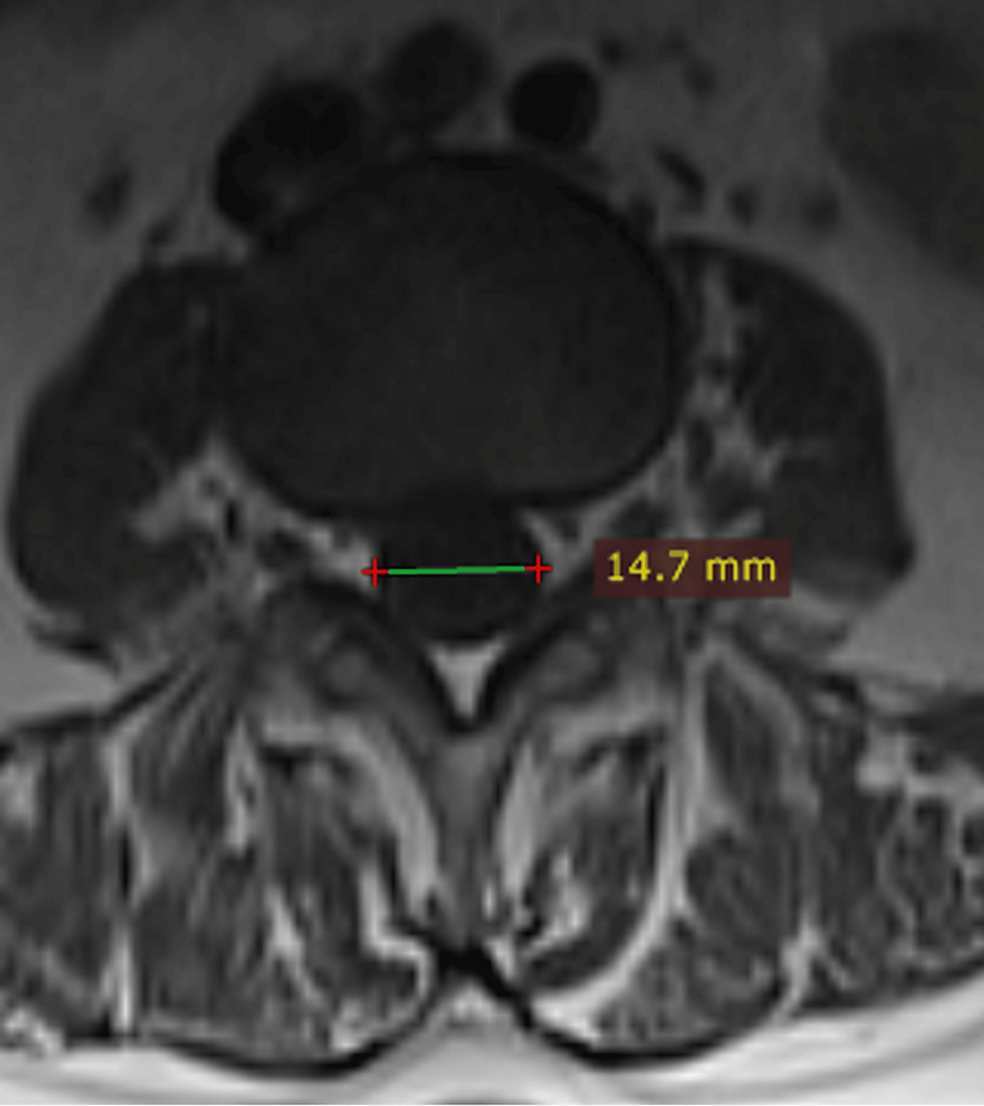
Transverse diameter of the vertebral canal in the axial plane

The transverse diameter of the vertebral canal was measured horizontally at the middle of the vertebral canal at the intervertebral disc level in the axial section (Figure 2).

**Figure 3:**
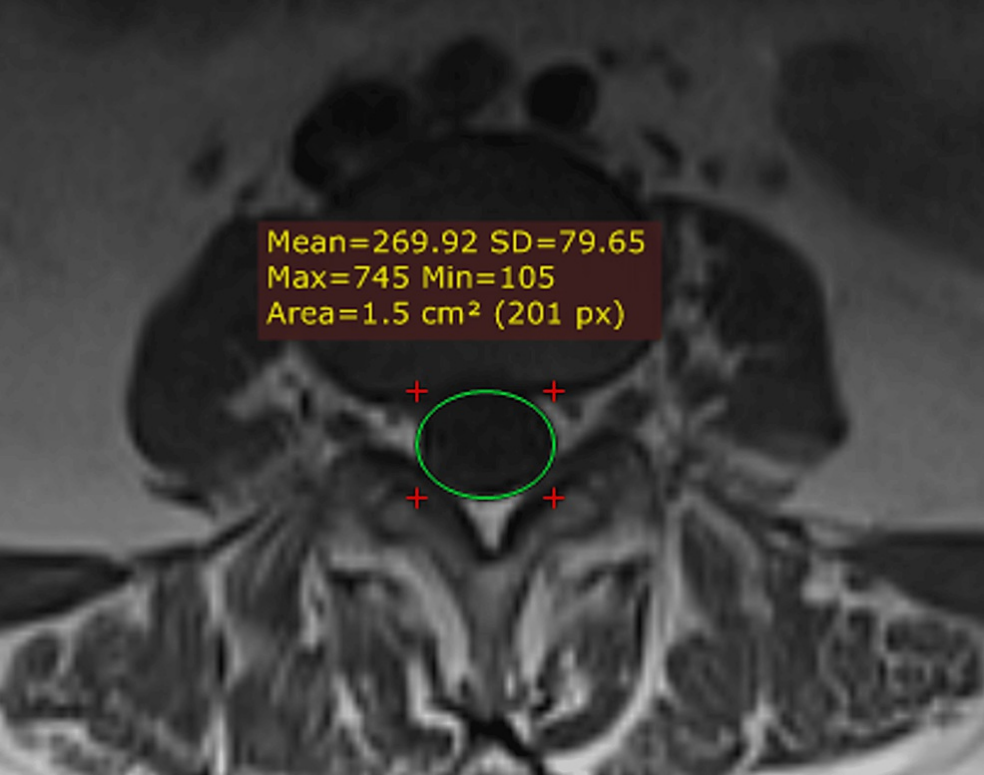
Thecal sac area of the vertebral canal in the axial plane SD: standard deviation

The thecal sac area of the vertebral canal was measured at the intervertebral disc level using a closed polygon and computed measurement software in the axial section (Figure 3).

The mean, range, and standard deviation were calculated for cases and controls separately. Statistical evaluation was done in both the cases and controls, gender-wise and age-wise. Student's t-tests were applied to know the significance of the difference between the means at each vertebral level in males and females and also in cases and controls. A p value of less than 0.05 was considered to be statistically significant. Age-wise analysis was done by categorizing the data into three age groups: group 1 (21-40 years), group 2 (41-60 years), and group 3 (61-80 years). The analysis of variance (ANOVA) was applied to test the significance of the difference between different age groups at all levels of lumbar vertebrae in the cases and controls.

## Results

Cases (subjects with low back pain)

The anteroposterior diameter of the lumbar vertebral canal decreased from L1 to L3 vertebra and then increased from L4 to L5 vertebra. Thus, the L3 vertebral canal had the minimum anteroposterior diameter among the lumbar vertebrae.

The transverse diameters of the vertebral canal decreased from L1-L2 to L5-S1 levels. Thus, the L5-S1 vertebral canal had the minimum transverse diameter among the different vertebral levels.

The thecal sac area of the vertebral canal decreased from L1-L2 to L4-L5 levels, and then, it increased at L5-S1 levels. Thus, the L4-L5 level vertebral canal had the smallest thecal sac area among the abovementioned vertebral levels (Table 1).

**Table 1:** Anteroposterior diameter (mm), transverse diameter (mm), and thecal sac area (cm2) of the vertebral canal at various levels in the subjects with low back pain SD: standard deviation

Level	Mean	Range	SD
Anteroposterior diameter (mm) of the vertebral canal			
L1	14.42	11.70-17.60	1.25
L2	14.09	10.10-16.90	1.32
L3	13.44	7.70-16.60	1.75
L4	13.63	9.20-16.80	1.75
L5	13.79	7.70-22.40	2.65
Transverse diameter (mm) of the vertebral canal			
L1-L2	17.36	8.90-22.60	2.59
L2-L3	16.43	11.10-20.10	2.23
L3-L4	14.58	8.20-18.90	2.57
L4-L5	13.07	7.10-18.50	3.19
L5-S1	12.97	5.90-18.90	2.99
Thecal sac area (cm^2^) of the vertebral canal			
L1-L2	1.77	0.80-2.80	0.37
L2-L3	1.62	0.90-2.80	0.32
L3-L4	1.32	0.60-2.70	0.38
L4-L5	1.10	0.40-2.60	0.41
L5-S1	1.28	0.40-2.80	0.40

On gender-wise evaluation, it was found that the anteroposterior diameter of the lumbar vertebral canal decreased from L1 to L3 level and then increased from L4 to L5 level in both males and females. Thus, the L3 vertebral canal had the minimum anteroposterior diameter among the lumbar vertebrae. However, no statistically significant difference was observed between males and females at any lumbar vertebral level.

The transverse diameter of the vertebral canal decreased from L1-L2 to L5-S1 levels in both males and females. Thus, the L5-S1 vertebral canal had the minimum transverse diameter among different vertebral levels. However, no statistically significant difference was observed between males and females at any lumbar vertebral level.

The thecal sac area of the vertebral canal decreased from L1-L2 to L4-L5 levels, and then, it increased at the L5-S1 levels in both males and females. Thus, the L4-L5 vertebral canal had the smallest thecal sac area among the various vertebral levels studied. However, no statistically significant difference was observed between males and females at any vertebral level studied (Table 2).

**Table 2:** Anteroposterior diameter (mm), transverse diameter (mm), and thecal sac area (cm2) of the vertebral canal in males and females at various levels in the subjects with low back pain Statistical test for significance: unpaired t-test SD: standard deviation

Level	Male (n=49)			Female (n=51)			p value
	Mean	Range	SD	Mean	Range	SD	
Anteroposterior diameter (mm) of the vertebral canal							
L1	14.53	12.20-17.60	1.25	14.32	11.70-16.70	1.25	0.283
L2	13.98	10.10-16.20	1.28	14.19	11.70-16.90	1.35	0.497
L3	13.36	7.70-16.60	1.80	13.51	10.30-16.50	1.71	0.815
L4	13.59	10.70-16.80	1.63	13.67	9.20-16.70	1.88	0.839
L5	13.88	7.70-22.40	3.18	13.70	9.20-17.50	2.03	0.805
Transverse diameter (mm) of the vertebral canal							
L1-L2	17.58	11.50-22.60	2.50	17.15	8.90-21.10	2.68	0.369
L2-L3	16.50	11.10-20.10	2.39	16.36	11.10-19.70	2.09	0.604
L3-L4	14.93	8.20-18.90	2.78	14.24	9.60-18.60	2.34	0.230
L4-L5	13.19	7.40-18.50	3.13	13.21	7.10-17.90	2.72	0.924
L5-S1	12.96	5.90-17.90	3.34	12.98	6.30-18.90	2.64	0.971
Thecal sac area (cm^2^) of the vertebral canal							
L1-L2	1.76	1.20-2.60	0.37	1.78	0.80-2.80	0.37	0.451
L2-L3	1.59	1.10-2.50	0.32	1.65	0.90-2.80	0.32	0.342
L3-L4	1.27	0.60-2.50	0.39	1.38	0.80-2.70	0.36	0.402
L4-L5	1.12	0.40-.2.60	0.43	1.08	0.50-1.90	0.39	0.827
L5-S1	1.30	0.40-2.80	0.49	1.26	0.80-2.00	0.27	0.195

Age-wise analysis showed no statistically significant difference in the anteroposterior diameter between the different age groups at all levels of lumbar vertebrae except L3.

There was no statistically significant difference in the transverse diameter between the different age groups at all levels of the vertebral canal except L4-L5 and L5-S1 levels.

There was no statistically significant difference in the thecal sac area between all the age groups at any vertebral level except at L4-L5 and L5-S1 levels (Table 3).

**Table 3:** Anteroposterior diameter (mm), transverse diameter (mm), and thecal sac area (cm2) of the vertebral canal in different age groups at various levels in the subjects with low back pain Statistical test for significance: ANOVA SD, standard deviation; ANOVA, analysis of variance

Level	Age: 21-40 years		Age: 41-60 years		Age: 61-80 years		p value
	Mean	SD	Mean	SD	Mean	SD	
Anteroposterior diameter (mm) of the vertebral canal							
L1	14.39	1.64	14.46	1.21	14.37	0.92	0.946
L2	14.44	1.25	13.98	1.34	14.02	1.32	0.372
L3	14.06	1.64	13.01	1.83	13.84	1.42	0.024
L4	13.72	1.93	13.64	1.68	13.52	1.81	0.926
L5	13.30	2.78	14.25	2.73	13.20	2.21	0.169
Transverse diameter (mm) of the vertebral canal							
L1-L2	16.71	3.27	17.47	2.21	17.70	2.71	0.387
L2-L3	16.64	2.15	16.49	2.25	16.10	2.31	0.696
L3-L4	15.25	2.06	14.73	2.59	13.62	2.78	0.080
L4-L5	13.99	2.87	13.76	2.65	11.24	2.75	<0.001
L5-S1	11.83	3.43	13.67	2.82	12.43	2.57	0.028
Thecal sac area (cm^2^) of the vertebral canal							
L1-L2	1.76	0.43	1.76	0.29	1.81	0.37	0.862
L2-L3	1.70	0.42	1.60	0.27	1.59	0.31	0.426
L3-L4	1.46	0.54	1.31	0.33	1.21	0.39	0.081
L4-L5	1.22	0.41	1.16	0.40	0.87	0.38	0.004
L5-S1	1.23	0.45	1.38	0.37	1.12	0.36	0.019

Controls (subjects without low back pain)

The anteroposterior diameter of the lumbar vertebral canal decreased from L1 to L3 vertebra and then increased from L4 to L5 vertebra. Thus, the L3 vertebral canal had the minimum anteroposterior diameter among the lumbar vertebrae.

The transverse diameter of the vertebral canal decreased from L1-L2 to L5-S1 levels. Thus, the L5-S1 vertebral canal had the minimum transverse diameter among the different vertebral levels.

The thecal sac area of the vertebral canal decreased from L1-L2 to L4-L5 levels and then increased at L5-S1 level. Thus, the L4-L5 vertebral canal had the smallest thecal sac area among the studied levels (Table 4).

**Table 4:** Anteroposterior diameter (mm), transverse diameter (mm), and thecal sac area (cm2) of the vertebral canal at various levels in the control subjects without low back pain SD: standard deviation

Level	Mean	Range	SD
Anteroposterior diameter (mm) of the vertebral canal			
L1	15.26	11.60-18.10	1.60
L2	15.16	11.60-19.00	1.67
L3	14.71	12.00-17.50	1.30
L4	14.68	12.00-17.90	1.36
L5	15.28	11.80-22.10	1.97
Transverse diameter (mm) of the vertebral canal			
L1-L2	18.71	15.40-23.90	2.16
L2-L3	18.11	14.60-23.60	2.15
L3-L4	16.87	13.10-22.10	2.10
L4-L5	15.87	11.30-21.70	2.36
L5-S1	14.98	8.20-19.50	2.18
Thecal sac area (cm^2^) of the vertebral canal			
L1-L2	2.05	1.50-3.20	0.40
L2-L3	1.95	1.40-3.20	0.39
L3-L4	1.80	1.40-2.70	0.34
L4-L5	1.72	1.40-3.00	0.32
L5-S1	1.73	1.40-2.60	0.26

On gender-wise evaluation, it was found that the anteroposterior diameter of the vertebral canal decreased from L1 to L3 level and then increased from L4 to L5 level in males. It decreased from L1 to L4 level and then increased at L5 level in females. However, no statistically significant difference was observed between males and females at all levels of lumbar vertebrae except at L1 and L5 levels.

The transverse diameter of the lumbar vertebral canal decreased from L1-L2 to L5-S1 levels in both males and females. Thus, the L5-S1 vertebral canal had the minimum transverse diameter among the different vertebral levels. It was also seen that the transverse diameter of the vertebral canal was greater in males than in females. A statistically significant difference was observed between males and females at all levels except at the L5-S1 level.

The thecal sac area of the vertebral canal in males decreased from L1-L2 to L4-L5 levels and then increased at L5-S1 level in both males and females. Thus, the L4-L5 vertebral canal had the smallest thecal sac area among the various vertebral levels studied. However, no statistically significant difference was observed between males and females at any vertebral level studied (Table 5).

**Table 5:** Anteroposterior diameter (mm), transverse diameter (mm), and thecal sac area (cm2) of the vertebral canal in males and females at various levels in the control subjects without low back pain Statistical test for significance: unpaired t-test SD: standard deviation

Level	Male (n=53)			Female (n=47)			p value
	Mean	Range	SD	Mean	Range	SD	
Anteroposterior diameter (mm) of the vertebral canal							
L1	15.54	11.60-17.90	1.59	14.95	12.00-18.10	1.58	0.018
L2	15.43	11.60-19.00	1.87	14.86	12.50-17.30	1.37	0.053
L3	14.84	12.00-17.50	1.45	14.56	12.00-16.60	1.09	0.216
L4	14.94	12.50-17.90	1.25	14.39	12.00-17.00	1.43	0.066
L5	15.62	12.50-17.30	1.20	14.89	11.80-22.10	2.53	0.048
Transverse diameter (mm) of the vertebral canal							
L1-L2	19.18	16.10-23.90	2.22	18.17	15.40-23.20	1.98	0.020
L2-L3	18.68	15.00-23.60	2.17	17.48	14.60-22.30	1.96	0.007
L3-L4	17.35	13.5022.10	2.29	16.33	13.10-20.40	1.73	0.010
L4-L5	16.36	12.40-21.70	2.72	15.33	11.30-18.10	1.75	0.027
L5-S1	15.34	12.00-17.70	1.54	14.89	8.20-22.10	2.69	0.083
Thecal sac area (cm^2^) of the vertebral canal							
L1-L2	2.10	1.50-3.20	0.40	1.99	1.50-3.20	0.40	0.644
L2-L3	2.00	1.40-3.20	0.39	1.90	1.40-2.90	0.37	0.815
L3-L4	1.85	1.40-2.70	0.35	1.76	1.40-2.70	0.33	0.318
L4-L5	1.77	1.40-3.00	0.37	1.67	1.40-2.30	0.23	0.140
L5-S1	1.78	1.40-2.60	0.29	1.68	1.40-2.20	0.22	0.145

Age-wise analysis showed no statistically significant difference in the anteroposterior diameter between the different age groups at all levels of lumbar vertebrae.

There was no statistically significant difference in the transverse diameter between the different age groups at all levels of the vertebral canal except at the L5-S1 level.

There was a statistically significant difference in the thecal sac area between all age groups at L3-L4, L4-L5, and L5-S1 levels (Table 6).

**Table 6:** Anteroposterior diameter (mm), transverse diameter (mm), and thecal sac area (cm2) of the vertebral canal in different age groups at various levels in the control subjects without low back pain Statistical test for significance: ANOVA SD, standard deviation; ANOVA, analysis of variance

Level	Age: 21-40 years		Age: 41-60 years		Age: 61-80 years		p value
	Mean	SD	Mean	SD	Mean	SD	
Anteroposterior diameter (mm) of the vertebral canal							
L1	15.35	1.63	15.25	1.64	14.43	1.03	0.415
L2	15.14	1.57	15.41	1.84	13.87	0.91	0.109
L3	14.81	1.36	14.61	1.27	14.30	0.71	0.552
L4	14.76	1.53	14.49	1.13	15.12	0.79	0.474
L5	15.49	2.25	14.91	1.52	15.47	1.06	0.370
Transverse diameter (mm) of the vertebral canal							
L1-L2	18.73	1.91	18.71	2.61	18.52	1.65	0.975
L2-L3	17.91	1.89	18.40	2.58	18.40	1.78	0.538
L3-L4	16.70	1.93	16.95	2.41	18.00	1.57	0.343
L4-L5	15.91	2.41	15.56	2.12	17.43	3.01	0.195
L5-S1	15.53	1.98	14.00	2.28	15.50	1.52	0.003
Thecal sac area (cm^2^) of the vertebral canal							
L1-L2	1.98	0.31	2.12	0.51	2.23	0.29	0.137
L2-L3	1.91	0.32	1.98	0.48	2.20	0.21	0.172
L3-L4	1.77	0.30	1.80	0.38	2.15	0.23	0.032
L4-L5	1.72	0.31	1.65	0.29	2.07	0.41	0.018
L5-S1	1.73	0.24	1.66	0.22	2.12	0.40	<0.001

The anteroposterior diameter of the vertebral canal was less at each level in the subjects with low back pain than in controls, and the difference was statistically significant. The transverse diameter of the vertebral canal was found to be smaller in cases compared to controls, with a statistically significant difference at each level studied. The thecal sac area of the lumbar vertebral canal was less at each vertebral level evaluated in the subjects with low back pain compared to controls. The difference was statistically significant (Table 7).

**Table 7:** Anteroposterior diameter (mm), transverse diameter (mm), and thecal sac area (cm2) of the vertebral canal in cases and controls at various levels Statistical test for significance: unpaired t-test SD: standard deviation

Level	Cases			Control			p value
	Mean	Range	SD	Mean	Range	SD	
Anteroposterior diameter (mm) of the vertebral canal							
L1	14.42	11.70-17.60	1.25	15.26	11.60-18.10	1.60	0.001
L2	14.09	10.10-16.90	1.32	15.16	11.60-19.00	1.67	0.009
L3	13.44	7.70-16.60	1.75	14.71	12.00-17.50	1.30	0.006
L4	13.63	9.20-16.80	1.75	14.68	12.00-17.90	1.36	0.005
L5	13.79	7.70-22.40	2.65	15.28	11.80-22.10	1.97	0.004
Transverse diameter (mm) of the vertebral canal							
L1-L2	17.36	8.90-22.60	2.59	18.71	15.40-23.90	2.16	0.009
L2-L3	16.43	11.10-20.10	2.23	18.11	14.60-23.60	2.15	0.004
L3-L4	14.58	8.20-18.90	2.57	16.87	13.10-22.10	2.10	0.004
L4-L5	13.07	7.10-18.50	3.19	15.87	11.30-21.70	2.36	0.001
L5-S1	12.97	5.90-18.90	2.99	14.98	8.20-19.50	2.18	0.001
Thecal sac area (cm^2^) of the vertebral canal							
L1-L2	1.77	0.80-2.80	0.37	2.05	1.50-3.20	0.40	0.001
L2-L3	1.62	0.90-2.80	0.32	1.95	1.40-3.20	0.39	<0.001
L3-L4	1.32	0.60-2.70	0.38	1.80	1.40-2.70	0.34	<0.001
L4-L5	1.10	0.40-2.60	0.41	1.72	1.40-3.00	0.32	0.006
L5-S1	1.28	0.40-2.80	0.40	1.73	1.40-2.60	0.26	<0.001

## Discussion

The anteroposterior diameter of the vertebral canal was less at each level in the subjects with low back pain compared to controls. The transverse diameter of the vertebral canal was found to be smaller in cases compared to controls, with a statistically significant difference at each level studied. The thecal sac area of the vertebral canal was found to be less at each vertebral level studied in the subjects with low back pain compared to controls.

In their study, Ahmad et al. performed MRI scans of 59 human subjects comprising 43 cases and 16 controls and found the mean values of the anteroposterior diameter of the vertebral canal to be 14.38, 14.11, 12.91, 12.26, and 11.00 in cases and 15.71, 15.21, 14.78, 14.64, and 13.71 in controls at L1, L2, L3, L4, and L5 levels, respectively. Furthermore, the difference in the anteroposterior diameter of the vertebral canal was statistically significant at L3, L4, and L5 levels between cases and controls [3]. The narrowing of the lumbar spinal canal has been reported in earlier cases of radicular syndrome [11].

El-Rakhawy et al. performed a study on plain X-rays of 200 controls comprising 100 males and 100 females and on the lumbar part of the vertebral column of 20 adult skeletons. They found that the mean values of the anteroposterior diameter of the lumbar vertebral canal were 13.1, 14.6, 12.3, 11.7, and 9.9 in males and 13.2, 13.4, 11.5, 11.0, and 9.5 in females on X-rays and 14.9, 15.0, 13.4, 15.4, and 15.6 on dry bones at L1, L2, L3, L4, and L5 levels, respectively [12]. However, when applied to soft tissue analysis on magnetic resonance imaging, traditional imaging measurements of lumbar vertebral canal diameters may not correlate [13].

The values obtained by Ahmad et al. [3] and El-Rakhawy et al. [12] in their study correlate with the present study's findings except at the L5 level, which was on the lower side. El-Rakhawy et al. highlighted that the lumbar vertebral canal was narrower among Egyptian subjects [12].

In their study, Pawar et al. performed MRI scans of 60 human subjects comprising 30 cases and 30 controls and found the mean values of the anteroposterior diameter of the lumbar vertebral canal to be 11.89, 11.95, 12.02, 10.60, and 9.05 in cases and 11.85, 12.27, 12.73, 12.98, and 13.11 in controls at L1, L2, L3, L4, and L5 levels, respectively. Furthermore, the difference in the lumbar vertebral canal's anteroposterior diameter was statistically significant, with a p value of <0.001 at L4 and L5 levels between cases and controls [9]. Singh et al.'s study (2016) reported that spinal canal anteroposterior diameters are larger in asymptomatic individuals than in low back pain patients. The authors suggested that spinal canal narrowing makes an individual susceptible to the compression of the cord and the eventual development of neurological signs and symptoms [14]. Dora et al. have also highlighted the significance of measuring spinal canal dimensions in discriminating asymptomatic disc herniations from symptomatic individuals [15]. In a recent study, Natalia et al. (2020) demonstrated a method that neuroradiologists may use to diagnose lumbar spinal stenosis on MRI with the help of anteroposterior diameter measurement [16].

Steurer et al. reviewed the radiological criteria reported for central stenosis. They found that the anteroposterior diameter and the cross-sectional area of the spinal canal were the commonly employed criteria [17].

The limitations of our study include a cross-sectional study design that does not give an idea about the progression of spinal stenosis and its correlation with low back pain symptoms. A larger sample size must be needed to generalize the study results to the larger population. Future large sample and prospective studies are required to devise methods for early diagnosis and help in the medical and surgical management of the pathology associated with low back pain.

## Conclusions

The study results give comparative data of the lumbar vertebral anteroposterior diameter, transverse diameter, and thecal sac area among the symptomatic low back pain patients and control subjects without low back pain. The MRI reflected decreased anteroposterior diameter, transverse diameter, and thecal sac area of the lumbar vertebral canal among symptomatic low back pain patients. The study results indicate that patients with low back pain have a higher probability of lumbar vertebral canal stenosis that can be confirmed by MRI, so early diagnosis and better management are made possible.
